# Tumor cell secretion of soluble factor(s) for specific immunosuppression

**DOI:** 10.1038/srep08913

**Published:** 2015-03-09

**Authors:** Arihiro Kano

**Affiliations:** 1 Institute for Materials Chemistry and Engineering, Kyushu University

## Abstract

Studies of tumor models using syngeneic transplantation have advanced our understanding of tumor immunity, including both immune surveillance and evasion. Murine mammary carcinoma 4T1 cells secrete immunosuppressive soluble factors as demonstrated in splenocyte culture. Cultured primary splenocytes secrete IFN-γ, which was strikingly elevated when the cells were isolated from 4T1 tumor-bearing mice. The secretion of IFN-γ peaked a week after 4T1 inoculation and then declined. This reduction may be due to the relatively decreased lymphocytes and increased granulocytes in a spleen accompanied by splenomegaly with time after the 4T1 inoculation. IFN-γ production was further suppressed with the addition of the conditioned media from 4T1 cells to the splenocyte culture. This suppressive effect was more evident in the splenocytes isolated from mice that had 4T1 tumors for a longer period of time and was not observed in the conditioned medium either from CT26 cells or with splenocytes isolated from CT26 tumor-bearing mice. These results suggest that the IFN-γ suppression is 4T1 tumor-specific. The soluble factor(s) in the 4T1-conditioned media was a protein between 10 to 100 kDa. The cytokine tip assay demonstrated several known cytokines that negatively regulate immune responses and may be candidates for this immunosuppression activity.

The concept of cancer immune surveillance, originally hypothesized by Burnet^1^ and Thomas^2^, indicates that immune cells recognize and eliminate continuously arising nascent transformed cells (reviewed in Ref. 3). However, tumors evade immune surveillance by various means (reviewed in Refs. 4,5,6,7,8,9,10), such as loss or down-regulation of tumor antigens and/or HLA class I antigens, defective death receptor signaling, lack of costimulatory molecules, production of immunosuppressive cytokines, and induction of immunosuppresive T cells and other suppressor cells. Eventually, tumor cells acquire metastatic ability. Dunn et al. later expanded the concept and proposed that cancer immunoediting, which involves immune selective pressure modifies the immunogenic phenotypes of tumors that eventually form in immunocompetent hosts^11^.

Syngeneic transplantation of tumor cells is a simple and easy method to evaluate tumor growth and immunity. The 4T1 cell line was derived from a spontaneously-arising mammary tumor from a BALB/c mouse. Its metastatic properties have been characterized^12^,^13^. In this cancer model, a leukemoid reaction and splenomegaly, which was characterized by massive granulocytic infiltration, were reported^14^,^15^. Myeloid-derived suppressor cells (MDSCs) are a heterogeneous population of immature myeloid cells that express both CD11b and Gr1 and cause T cell dysfunction in tumor-bearing mice (reviewed in Ref. 16). In a 4T1-transplanted BALB/c mouse, the actual number of lymphocytes remains constant, while a striking increase in immature myeloid cells in the bone marrow, spleen, and peripheral blood has been reported^14^. It is of interest that a reduction in MDSCs results in delayed tumor progression^15^,^17^.

Interferon-γ (IFN-γ) is a pleiotropic cytokine that is produced principally by both natural killer (NK) cells and T lymphocytes and plays a role in inhibiting tumor development, growth, and metastasis (reviewed in Refs. 18, 19). It has been demonstrated that 4T1 tumors inoculated in IFN-γ-deficient mice grow slightly faster and metastasize more readily^20^,^21^, suggesting an important role of IFN-γ in immunity for blocking tumor progression. In this paper, secretion of IFN-γ in splenocyte culture, using splenocytes harvested from 4T1 tumor-bearing mice, was examined. The secretion of IFN-γ by these harvested cells in culture was transiently increased at approximately a week after tumor transplantation and then reduced to almost an undetectable level in three weeks. These novel findings may reflect an *in vivo* immunological status. When the harvested splenocytes were cultured with the conditioned media of 4T1 cells, the production of IFN-γ, but not that of TNF-α, was suppressed, and the level of suppression increased with the length of time after the transplantation. The suppression of the immune response by the conditioned media of CT26 colon tumor cells was not significant relative to the increased activity observed with 4T1 tumor cells. These results suggest that 4T1 tumor cells secrete soluble factors that have specific immune suppression activity.

## Methods

### Cell culture and animal studies

Murine mammary carcinoma 4T1 cells and colon tumor CT26 cells were purchased from American Type Culture Collection (ATCC) (Manassas, VA, USA), and maintained with either RPMI-1640 or D-MEM (Wako Pure Chemical Industries, Ltd., Osaka, Japan) supplemented with 10% heat-inactivated fetal bovine serum in a 5% CO_2_ atmosphere at 37°C. Balb/c mice were purchased from Kyudo Co. Ltd. (Tosu, Japan) and used at the age of 9–20 weeks. All animal experiments were carried out according to the guidelines for proper conduct of animal experiments published by the Science Council of Japan. All experimental protocols were approved by the Ethics Committee and the Animal Care and Use Committee of Kyushu University. The animals were inoculated with 1 × 10^6^ 4T1 or CT26 cells subcutaneously in the dorsal region. Splenocytes were prepared from a spleen of either an untreated or tumor-inoculated Balb/c mouse as below. The harvested spleen was minced well and further dissociated by passing several times through a 10-ml syringe equipped with a 19-G needle. The suspension was centrifuged, and the pellet was washed with RPMI-1640. The washed pellet was resuspended with ACK lysing buffer (Lonza, Walkersville, MD, USA) and incubated for 5 min. After centrifugation, the pellet was washed and resuspended in RPMI-1640. The cell suspension was placed on a solution of Lympholyte-M (Cedarlane Laboratories Ltd., Burlington, Ontario, Canada) and centrifuged for 15 min at 800 × g. The cells on the top of Lympholyte-M were recovered and centrifuged. The centrifuged pellet was resuspended with RPMI-1640 and centrifuged again. The pelleted cells were recovered and suspended in RPMI-1640 supplemented with 10% fetal bovine serum and 100-fold diluted PenStrep solution (GIBCO, Grand Island, NY, USA). The recovered splenocytes were seeded at one million cells per well in a 96-well culture plate (Thermo Fisher Science Inc., Waltham, MA, USA) and cultured for 2 to 3 days with or without 10% tumor cell-conditioned medium. The tumor cells were cultured to subconfluence, and then the medium was replaced freshly. After three days, the medium was recovered and filtered through a sterile 0.22 μm syringe filter to prepare tumor-conditioned medium. For the co-culture experiment, one million splenocytes were mixed with the indicated number of trypsinized 4T1 cells and seeded in a 96-well culture plate with 150 μl of RPMI-1640 supplemented with 10% serum. The medium was recovered after two days and subjected to cytokine analysis.

### Enzyme-linked immunosorbent assay (ELISA)

Quantification of IFN-γ, TNF-α, IL-10, and TGF-β in the tissue-conditioned media was performed by ELISA (R&D Systems Inc., Minneapolis, MN, USA), according to the manufacturer's instructions.

### Quantitative RT-PCR

Total RNA was purified with TRIzol reagent (Life Technologies Japan Ltd, Tokyo, Japan) according to the manufacture's instructions. Single-stranded cDNA was synthesized from total RNA using High Capacity cDNA Reverse Transcription Kits (Life Technologies Japan Ltd), according to the manufacture's instructions. The synthesized cDNA was subjected to the quantitative PCR using TaqMan Gene Expression Assays for IFN-γ, Thy1, F4/80, and GAPDH (Life Technologies Japan Ltd). The messenger RNA expression levels were calculated from the cycle threshold (*C*_t_) values and normalized with the expression levels of GAPDH. The normalized expression levels were further demonstrated as the fold induction compared to the initial levels of each mRNA such as of 3 h culture.

### Cytokine tip assay

The biotin label-based cytokine tip assay (Cat#: AAM-BLG-1, RayBiotech, Inc., Norcross, GA, USA) was performed according to the manufacturer's instructions.

### Statistical analysis

Statistical evaluation of differences between the experimental groups was conducted using analysis of variance and two-tailed unpaired Student's t tests. *P* values < 0.05 were considered statistically significant. All experiments were performed at least three times.

## Results

### Production of IFN-γ and TNF-α in splenocyte culture

**Figure 1:**
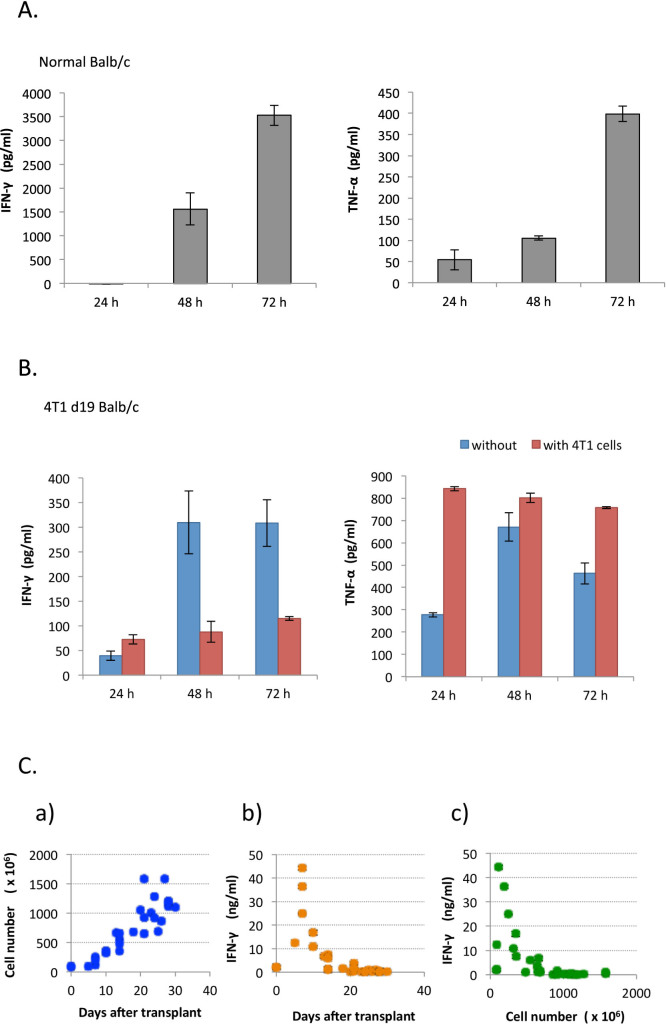
Secretion of IFN-γ and TNF-α by splenocytes. (A). Splenocytes isolated from an untreated Balb/c mouse were cultured for the indicated time, and IFN-γ and TNF-α in the medium were measured by a sandwich ELISA. Splenocyte culture was performed in triplicate and the results were expressed as mean ± standard deviation (SD). Typical results are shown. (B). Splenocytes isolated from 4T1 tumor-bearing mice (19-day after 4T1 cell inoculation) were cultured with or without 20,000 cells of 4T1 for the indicated time, and IFN-γ and TNF-α were measured. Splenocyte culture was performed in triplicate and the results were expressed as mean ± SD. Typical results are shown. (C). Correlations between isolated cell number from a spleen (a), secretion of IFN-γ (b) and the day after 4T1 cell inoculation, with secretion of IFN-γ and isolated cell number from a spleen (c) are shown. Each circular symbol corresponds to a mouse.

The splenocytes isolated from a Balb/c mouse were seeded on a 96-well culture plate. After the indicated time, the levels of IFN-γ and TNF-α in the conditioned media were evaluated. The levels of both cytokines released into the splenocyte culture medium increased with time (Fig. 1A). When splenocytes isolated from 4T1-bearing mice were used, both cytokines were secreted at an earlier time point. However, the level of IFN-γ production was markedly reduced, while that of TNF-α was greatly increased (Fig. 1B). Massive splenomegaly was observed in the 4T1-bearing mice, which has been reported as due to granulocytic hyperplasia^14^. In this study, the total number of splenocytes isolated from 4T1-bearing mice gradually increased with time after 4T1 transplantation (Fig. 1C, a). Using a sandwich ELISA, the level of IFN-γ was found to be strikingly increased, more than 20-fold, at day 7 after the transplantation, and then reduced to almost undetectable levels by day 28 (Fig. 1C, b). The reduction of IFN-γ was negatively correlated with the isolated number of splenocytes after day 7 (Fig. 1C, c). DuPre et al. reported that the number of lymphocytes in a spleen remained constant, while that of granulocyte increased more than 40-fold^14^, thus, the relative number of lymphocytes was decreased. Similarly, Le et al. showed that the proliferation of T cells isolated from a spleen of the 4T1 tumor-bearing mouse were suppressed, which was recovered by MDSC depletion^15^. Thus, it is thought that the decreased IFN-γ production is due to the reduced relative number of lymphocytes and emerged MDSC in the spleen of the 4T1 tumor-bearing mouse.

### Co-culture with 4T1 cells and culture with 4T1-conditioned medium suppresses IFN-Υ secretion by splenocytes

**Figure 2:**
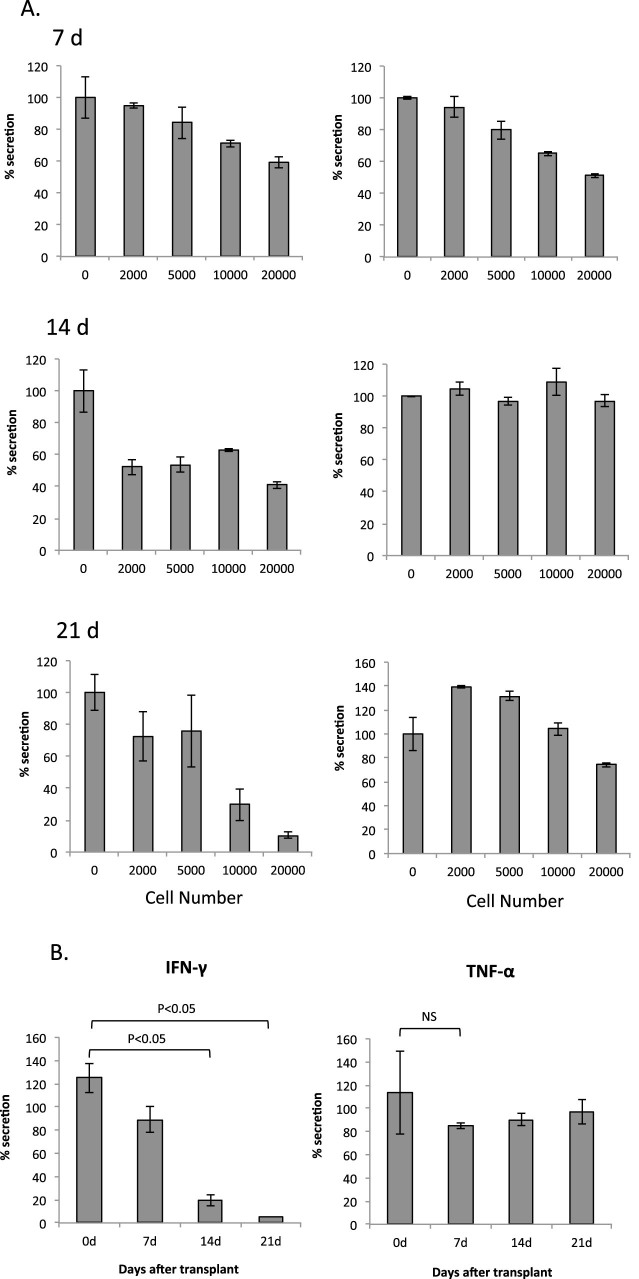
Suppression of IFN-γ secretion by splenocytes isolated from 4T1 tumor-bearing mice. (A). Splenocytes isolated on the indicated day after 4T1 inoculation were cultured with 4T1 cells, and IFN-γ and TNF-α in the culture medium were measured. Splenocyte culture was performed in triplicate and the results were expressed as mean ± SD. The data are expressed as the percentage of the findings with splenocytes alone. Typical results are shown. (B). Splenocytes isolated on the indicated day after 4T1 cell inoculation were cultured with the conditioned medium of 4T1 cells, and IFN-γ and TNF-α in the culture medium were measured. The data are expressed as the percentage of the findings without the conditioned medium. The splenocytes were isolated from three mice each, and the average is shown. The errors quoted are standard deviation from the mean. NS: No significance.

To investigate the activation of T cells that would target 4T1 cells, the splenocytes isolated from a 4T1 tumor-bearing mouse at the indicated day after tumor inoculation were cultured with 4T1 cells (Fig. 2A). Unexpectedly, the secretion of IFN-γ was suppressed by co-culture with 4T1 cells and this reduction was more notable in the splenocytes that were isolated from mice bearing 4T1 tumors for a longer time as compared to those mice carrying the tumors for shorter periods of time. In contrast, co-culture had no effect on TNF-α levels whether the splenocytes were from tumor-bearing mice or not. Next, the conditioned medium of 4T1 cells was used for splenocyte culture, and IFN-γ levels were examined. The secretion of IFN-γ was suppressed by the 10% conditioned medium, and the level of this suppression was also dependent on the length of time the mice had the tumors (Fig. 2B). The conditioned medium from 4T1 cells did not affect the levels of TNF-α secreted from any of the splenocytes.

### Regulation of IFN-γ in splenocyte culture

**Figure 3:**
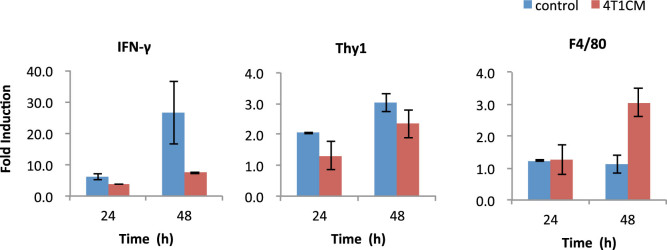
Quantitative RT-PCR in cultured splenocytes isolated from 4T1 tumor-bearing mice. The fold changes in mRNA expression of IFN-γ, Thy1, and F4/80 with or without 4T1-conditioned medium (4T1CM) compared with the initial levels (3 h culture) are shown. The quantitative RT-PCR was performed in triplicate and the results were expressed as mean ± SD. The splenocytes were isolated from three mice and typical results are shown.

The quantitative RT-PCR was performed in the cultured splenocytes isolated from the mouse bearing 4T1 tumor for three weeks (Fig. 3). The IFN-γ expression was upregulated several folds in 24 h and more than 20-fold in 48 h compared to the 3 h-cultured splenocytes, while it was suppressed substantially in 48 h culture with the 4T1-conditioned medium. The Thy1 expression, a T cell marker, was slightly reduced, however, there was no difference between 24 h and 48 h. Therefore it is unlikely that the reduction of T cell number is the reason for the IFN-γ suppression by 4T1-conditioned media. On the other hand, the expression of F4/80 was increased in 48 h culture with 4T1-conditioned media, suggesting monocyte proliferation. Taken together, these results suggest that the IFN-γ expression in splenocytes is upregulated in the mRNA level and this upregulation is suppressed by 4T1-conditioned media. The proliferated monocytes may inhibit the T cell activation.

### Specific suppression of IFN-Υ secretion for 4T1 cells

**Figure 4:**
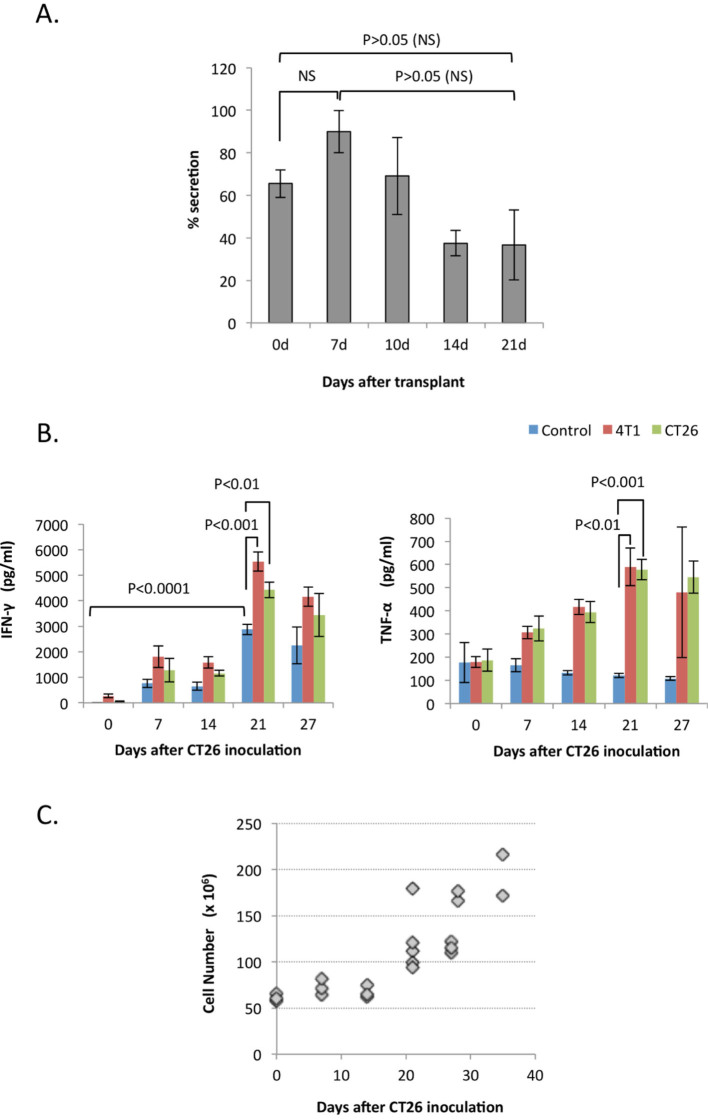
Effect of CT26-conditioned medium. (A). Splenocytes isolated on the indicated day after 4T1 cell inoculation were cultured with the conditioned medium of CT26 cells, and IFN-γ in the culture medium was measured. The data are expressed as the percentage of the findings without the conditioned medium. The splenocytes were isolated from three mice each and the average is shown. The errors quoted are standard deviation from the mean. NS: No significance (B). Splenocytes isolated on the indicated day after CT26 cell inoculation were cultured with or without the conditioned medium of either 4T1 or CT26 cells, and IFN-γ and TNF-α in the culture medium were measured. The splenocytes were isolated from three mice each and the average is shown. The errors quoted are standard deviation from the mean. (C). The total splenocyte number isolated from the CT26 tumor-bearing mice after the indicated day after the inoculation. Each symbol corresponds to a mouse.

The splenocytes isolated from 4T1-bearing mice were cultured with the CT26 colon tumor cell-conditioned medium and IFN-γ secretion was evaluated. The CT26-conditioned medium suppressed IFN-γ secretion by the splenocytes harvested from mice bearing the 4T1 tumor for two weeks or longer, however, the reduction in secretion was not as robust as that was seen with the 4T1-conditioned media (Fig. 4A). Next, splenocytes were isolated from CT26-bearing mice on the indicated days after transplant and cultured with either 4T1- or CT26-cell conditioned medium (Fig. 4B). The secretion of IFN-γ was markedly increased in splenocytes isolated from mice inoculated with CT26 tumors and the level reached a maximum in the cells isolated from the mice at 21-day after the tumor inoculation. Conditioned media either from 4T1 cells or CT26 cells slightly stimulated IFN-γ secretion at all times after the tumor transplant, and there was no significant difference between both media. The secretion of TNF-α was stimulated by both conditioned media and gradually increased up to day 21. It suggests that tumors have some influence on the spleen and/or on splenocytes, e.g. cell type in the spleen, activation of dendritic cells, etc., which are sensitive to some external stimulation *in vitro*. The number of the isolated splenocytes started to increase three weeks after the inoculation. It was roughly five-fold less than that was seen in the 4T1 inoculation (Fig. 4C). Collectively, the 4T1-conditioned medium specifically suppresses IFN-γ secretion by splenocytes isolated from 4T1-bearing mice.

### Characterization of the factors in 4T1-conditioned medium

**Figure 5:**
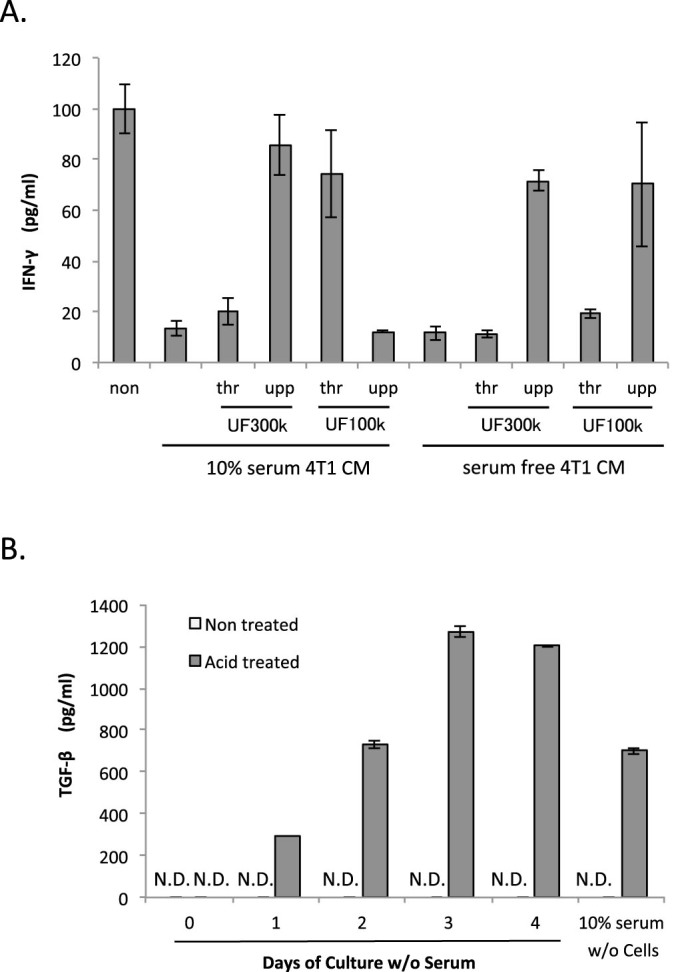
Characterization of the immunosuppressive activity in 4T1-conditioned medium. (A). Splenocytes isolated from a 4T1 tumor-bearing mouse were cultured with 4T1-conditioned medium fractionated with the indicated ultrafiltration, and IFN-γ in the culture medium was measured. (B). TGF-β in 4T1-conditioned medium cultured for the indicated days was measured. The samples were treated with or without acid. Splenocyte culture was performed in triplicate and the results were expressed as mean ± SD. Typical results are shown.

It was expected that the soluble factors in the conditioned medium played a key role in the observed reduction in IFN-γ secretion by splenocytes. At first, ultrafiltered fractions of the 4T1-conditioned medium were tested (Fig. 5A). The suppressive activity of the conditioned medium with 10% serum were observed in the fraction that passed through the 300 kDa and did not pass through the 100 kDa molecular weight cut-off (MWCO) filters. The activity of the medium without serum was observed in the fractions that passed through both the 300 kDa and 100 kDa filters (Fig. 5A) but not through the 10 kDa filters (data not shown). These results suggest that the immune suppressive soluble factor(s) is between 10 to 100 kDa molecular weight and are bound to serum proteins. Combined with the finding that the activity was heat-sensitive (data not shown), the factor(s) was predicted to be a protein.

Next, a sandwich ELISA was performed for IL-10 and TGF-β as both are considered immunosuppressive cytokines^22^,^23^,^24^,^25^. However, IL-10 was undetectable (at the level of picograms per milliliter). The secreted levels of TGF-β increased with time in culture but could only be detected in the acid-treated samples, suggesting that 4T1 cells secrete the latent form of TGF-β^26^ (Fig. 5B). Substantial amounts of TGF-β were also observed in the acid-treated 10% serum-containing medium. IFN-γ was secreted by splenocytes in 10% serum and mature murine TGF-β differs only in a single amino acid position from bovine, therefore, the 4T1-secreted TGF-β is likely not involved in the suppression of IFN-γ secretion.

### Cytokine Tip Assay on the conditioned media

Finally, the comprehensive biotin label-based cytokine tip assay was performed, which is able to detect 308 proteins semi-quantitatively. The detected proteins in 4T1-conditioned medium are listed in the order of relative fluorescent intensity in Table 1 and with the positive control, biotinylated IgG, as a reference. The strongest signal was obtained for MMP-3, a member of the matrix metalloproteinase family of extracellular proteinases. The correlation of MMPs with the presence of tumor metastasis has been demonstrated^27^. Fas, a plasma membrane receptor for inducing apoptosis on cells, was also detected at a level similar to that of MMP-3. Soluble Fas is thought to work as an antagonist for Fas ligand and an elevation of the soluble form has been reported in the serum of patients with bladder^28^, breast^29^, renal cell^30^, hepatocellular^31^, gynecological^32^, ovarian^33^, and prostate cancer^34^. A considerable signal for IL-10 was detected, however, the sandwich ELISA assay failed to detect it. The cytokine tip assay is based on biotin labeling of proteins and the specificity is achieved by a single antibody fixed on a glass slide. At present, it is unknown whether the results are due to cross-reactivity in the tip assay or to undetectable levels in the sandwich ELISA. TGF-β was also significantly detected probably in the latent form as seen in the sandwich ELISA. VEGF was also identified as abundant. VEGF is reported to be produced by almost all tumors and inhibits dendritic cell maturation, resulting in defective immune function in cancer^35^. VEGF may be involved in the observed IFN-γ suppression in the splenocyte culture. Several inflammatory cytokines were also detected in substantial amounts in the 4T1-conditioned medium, including IL-3, IL-17, IFN-γ, IL-12, IL-23, and IL-27. These are known inflammatory cytokines, and therefore not likely to be involved in the observed immunosuppression. Indeed, the sandwich ELISA failed to detect IFN-γ in the 4T1-conditioned medium (data not shown).

## Discussion

To better understand immune reactions in transplanted tumors, secretion of IFN-γ by cultured splenocytes isolated from tumor-bearing mice was measured. In this new assay, I found increased IFN-γ secretion in murine splenocyte culture medium. The secretion of IFN-γ was dependent on the cell density in culture, indicating the importance of the cell-cell interactions in this system. Although the splenocytes harvested from the mice at 19-day after 4T1 tumor inoculation secreted IFN-γ at an earlier time in culture, which was detected at 24 h, and reached a plateau at 48 h, the secreted amount was significantly decreased. This reduced level is probably due to the reduced relative number of T cells by a massive increment of granulocytes in the spleen and also due to the suppression of T cell activation by the emerged MDSCs. Indeed, Le et al. have observed the recovered proliferative activities of the splenic T cells *in vitro* by the MDSC depletion with anti Gr-1 antibody^15^. Unexpectedly, the secretion of IFN-γ was further suppressed by the conditioned medium of 4T1 cells and this suppression increased with time after tumor transplant. Furthermore, the suppressive effect of 4T1-conditioned medium was found to be greater than that observed with CT26-conditioned medium. Since the 4T1-derived active factor(s) was characterized as a protein(s) between 10 and 100 kDa, the cytokine tip assay was performed. IL-10 and TGF-β, which are already widely reported as immunosuppressive cytokines, were detected in abundance, but the sandwich ELISA did not detect these cytokines. The quantitative RT-PCR showed increased F4/80 expression suggesting monocyte proliferation in the splenocyte culture with 4T1-conditioned medium. One of the hypotheses is that tumor-derived soluble factors stimulate MDSCs and suppress T cell activation. G-CSF or GM-CSF may be a candidate for the observed immunosuppression. However, further verification of the tip assay findings is required.

Cellular senescence was originally described as normal primary cells that had a limited proliferative ability in culture^36^. Now it has been recognized that cellular senescence is also induced by oncogenes or chemotherapeutic drugs^37^. Recently, it was clearly shown that the oncogene-induced senescent cells are eliminated by a CD4^+^ T-cell-mediated adaptive immune response, designated as ‘senescence surveillance’ by the authors^38^. The report of the importance of IFN-γ and TNF-α for the induction of permanent growth arrest in cancers^39^ is of interest. The senescent cells secrete a number of factors that may initiate senescence surveillance, suggested in several papers^38^,^40^. The correlation of secreted factors by senescent cancer cells and T-cell activation and/or suppression are interesting topics. My observations, the secreted factor(s) from 4T1 tumor cells suppresses IFN-γ expression, may also correlate with ‘senescence surveillance’.

The emergence of cancer in a body influences the immune system, resulting in changes to immune cell constitution and activation status. These changes in immune cell types and amounts are often studied using either flow cytometry or isolated immune cells. Previously, I successfully evaluated the immune reaction by measuring IFN-γ in cultured splenocytes one day after stimulation with lipopolysaccharides (LPS)^41^. In the present study, splenocytes were cultured without LPS but for a longer time, two or three days. Although the levels of IFN-γ secretion vary with splenocyte density, age of the mice, and probably the environment of the mice, the conditioned media of 4T1 cells suppressed the levels significantly in splenocytes derived from tumor-bearing mice. It is unclear how IFN-γ secretion is regulated in splenocyte culture. However, this new culture method may contribute to the understanding of immune regulation by 4T1 tumors and by other tumor cell types.

Although the cytokine tip assay showed some known suppressive cytokines in the 4T1-conditioned media, further examination is required to identify the molecules that suppress IFN-γ production in this system. Weak suppression was also observed in the cells derived from CT26-bearing mice. It is not clear whether the same factor is active in both 4T1 cells and CT26 cells. The testing of additional tumor models in this system should be done and will likely yield important information on the immunosuppressive factor(s). The culture of splenocytes is a multi-cellular complex system. Therefore, the production of IFN-γ and its suppression, are complex and may involve a cascade of events between and within different cell types. Investigation of the immunosuppressive factors using this system may define a new aspect in the specific and/or nonspecific immunosuppression mediated by cancer cells.

## Author Contributions

A.K. wrote the manuscript and prepared all figures. The author reviewed the manuscript.

## Figures and Tables

**Table 1:** Proteins identified in the cytokine tip assay on 4T1-conditioned medium. The detected proteins are presented in order of abundance (strong fluorescence signal). The positive control, Positive 1a: biotinylated IgG, is shown as a reference

No.	Name	Fl. Int.	No.	Name	Fl. Int.	No.	Name	Fl. Int.	No.	Name	Fl. Int.
1	MMP-3	56253.0	31	TNF RII	10134.9	60	6Ckine	2131.1	90	IL-9	1022.3
2	Fas/TNFRSF6	56103.9	32	TRAIL R2/TNFRSF10B	9639.4	61	CD30 L	2125.9	91	CCL28	1015.2
3	CD11b	43313.1	33	Osteopontin	9614.1	62	BLC	2095.3	92	IL-3 R beta	1015.1
4	IL-3	41246.5	34	KC	6630.7	63	TIMP-1	2005.4	93	CXCR2/IL-8 RB	1003.8
5	SLPI	39782.4	35	IL-27	6547.1	64	HGF R	2005.1	94	GITR	994.2
6	IFN-gamma	36116.9	36	CTACK	5452.7	65	Fit-3 Ligand	1946.1	95	GFR alpha-2/GDNF R alpha-2	960.7
7	IL-12 p70	33909.0	37	Activin A	5232.2	66	FCrRIIB/CD32b	1913.0	96	MMP-12	853.2
8	G-CSF	32550.7	38	IL-1 alpha	5082.4	67	Artemin	1867.5	97	CCL1/I-309/TCA-3	823.5
9	MMP-9	30786.5	39	IL-23	4949.7	68	E-Selectin	1830.7	98	Cripto	799.0
10	IL-10	29890.7	40	MDC	4110.8	69	P-Selectin	1810.1	99	CCR7	765.2
11	IL-17	24631.9	41	FGF R3	4013.8	70	FAM3B	1750.9	100	PDGF C	671.3
12	IL-12 p40/p70	24488.0		**Positive 1a**	**3999.7**	71	Dkk-4	1637.9	101	GDF-8	615.4
13	IL-6	24463.7	42	IL-6 R	3981.0	72	IL-1 R4/ST2	1552.8	102	IL-17R	580.4
14	IL-5	23376.8	43	Dtk	3842.6	73	CCR6	1544.8	103	IFN-alpha/beta R1	574.4
15	IFN-beta	23320.9	44	CD14	3751.2	74	PDGF R beta	1506.7	104	GFR alpha-3/GDNF R alpha-3	573.7
16	GM-CSF	22409.7	45	Galectin-3	3716.3	75	Adiponectin/Acrp30	1457.7	105	EG-VEGF/PK1	556.5
17	TNF-alpha	21211.3	46	GDF-1	3630.5	76	IGFBP-5	1422.4	106	IGFBP-1	535.0
18	TGF-beta 1	20006.3	47	Activin C	3254.6	77	AR (Amphiregulin)	1393.7	107	CRG-2	482.0
19	MIP-3 alpha	19293.3	48	GITR Ligand/TNFSF18	3133.1	78	IL-4 R	1383.2	108	IL-17BR	461.0
20	VCAM-1	18788.1	49	Eotaxin-2	3111.8	79	IFN-gamma R1	1382.5	109	ICAM-2/CD102	442.7
21	PlGF-2	17725.3	50	CD27/TNFRSF7	2874.7	80	BCMA/TNFRSF17	1342.4	110	Fractalkine	401.6
22	Progranulin	16797.8	51	TIMP-2	2855.5	81	DR3/TNFRSF25	1333.2	111	CD40	340.6
23	TSLP	16476.2	52	IL-1 RII	2785.5	82	IGFBP-6	1331.7	112	Erythropoietin (EPO)	335.1
24	MCP-1	15309.5	53	IL-5 R alpha	2652.6	83	Angiopoietin-like 3	1313.5	113	Granzyme D	271.6
25	MMP-14/LEM-2	14159.6	54	GDF-3	2599.2	84	CD40 Ligand/TNFSF5	1298.8	114	IL-7 R alpha	250.1
26	LIF	13992.0	55	MMP-24/MT5-MMP	2583.0	85	EGF R	1248.6	115	Epigen	226.4
27	TNF RI/TNFRSF1A	13584.4	56	Granzyme B	2344.7	86	CCR10	1195.8	116	IFN-alpha/beta R2	178.3
28	VEGF	13184.7	57	CD30	2198.5	87	IL-2	1116.8	117	IL-15 R alpha	168.5
29	CXCL16	12707.3	58	MIG	2168.9	88	IL-7	1066.0	118	Dkk-3	162.9
30	VE-Cadherin	10426.4	59	Endoglin/CD105	2157.7	89	CD27 Ligand/TNFSF7	1062.7	119	Resistin	125.3
